# Physical activity and inactivity patterns in India – results from the ICMR-INDIAB study (Phase-1) [ICMR-INDIAB-5]

**DOI:** 10.1186/1479-5868-11-26

**Published:** 2014-02-26

**Authors:** Ranjit M Anjana, Rajendra Pradeepa, Ashok K Das, Mohan Deepa, Anil Bhansali, Shashank R Joshi, Prashant P Joshi, Vinay K Dhandhania, Paturi V Rao, Vasudevan Sudha, Radhakrishnan Subashini, Ranjit Unnikrishnan, Sri V Madhu, Tanvir Kaur, Viswanathan Mohan, Deepak K Shukla

**Affiliations:** 1 Department of Epidemiology & Diabetology, Madras Diabetes Research Foundation & Dr.Mohan’s Diabetes Specialities Centre, WHO Collaborating Centre for Noncommunicable Diseases Prevention and Control & IDF Centre of Education, 4, Conran Smith Road, Gopalapuram, Chennai 600 086, India; 2 Department of Endocrinology, Jawaharlal Institute of Post– Graduate Medical Education & Research, Puducherry, India; 3 Department of Endocrinology, Postgraduate Institute of Medical Education and Research, Chandigarh, India; 4 Department of Endocrinology, Lilavati Hospital, Mumbai, India; 5 Department of Medicine, Indira Gandhi Government Medical College, Nagpur, India; 6 Department of Diabetology, Diabetes Care Center, Ranchi, India; 7 Department of Endocrinology & Metabolism, Nizam’s Institute of Medical Sciences, Hyderabad, India; 8 Department of Medicine, University College of Medical Sciences and GTB Hospital, Delhi, India; 9 Department of Non Communicable Diseases, Indian Council of Medical Research, New Delhi, India

**Keywords:** Prevalence, Physical activity, INDIAB, India, South Asians, Asian Indians, Exercise, Sedentary, Diabetes

## Abstract

**Background:**

The rising prevalence of diabetes and obesity in India can be attributed, at least in part, to increasing levels of physical inactivity. However, there has been no nationwide survey in India on physical activity levels involving both the urban and rural areas in whole states of India. The aim of the present study was to assess physical activity patterns across India - as part of the Indian Council of Medical Research-India Diabetes (ICMR-INDIAB) study.

**Methods:**

Phase 1 of the ICMR-INDIAB study was conducted in four regions of India (Tamilnadu, Maharashtra, Jharkhand and Chandigarh representing the south, west, east and north of India respectively) with a combined population of 213 million people. Physical activity was assessed using the Global Physical Activity Questionnaire (GPAQ) in 14227 individuals aged ≥ 20 years [urban- 4,173; rural- 10,054], selected from the above regions using a stratified multistage design.

**Results:**

Of the 14227 individuals studied, 54.4% (n = 7737) were inactive (males: 41.7%), while 31.9% (n = 4537) (males: 58.3%) were active and 13.7% (n = 1953) (males: 61.3%) were highly active. Subjects were more inactive in urban, compared to rural, areas (65.0% vs. 50.0%; p < 0.001). Males were significantly more active than females (p < 0.001). Subjects in all four regions spent more active minutes at work than in the commuting and recreation domains. Absence of recreational activity was reported by 88.4%, 94.8%, 91.3% and 93.1% of the subjects in Chandigarh, Jharkhand, Maharashtra and Tamilnadu respectively. The percentage of individuals with no recreational activity increased with age (Trend *χ*^2^: 199.1, p < 0.001).

**Conclusions:**

The study shows that a large percentage of people in India are inactive with fewer than 10% engaging in recreational physical activity. Therefore, urgent steps need to be initiated to promote physical activity to stem the twin epidemics of diabetes and obesity in India.

## Background

The International Diabetes Federation estimates that more than 382 million people worldwide have diabetes as of 2013, and this number is projected to increase to 592 million by the year 2035 [1]. Low and middle income countries are expected to contribute to most of this increase with China and India alone contributing to 163.5 million individuals with diabetes globally [1].

The explosive increase in the prevalence of type 2 diabetes is due, in large measure, to the adoption of unhealthy lifestyle practices by individuals at risk of developing the disorder. Indeed, insufficient physical activity and unhealthy diets have emerged as two of the most important modifiable risk factors not only for type 2 diabetes, but for other chronic non communicable diseases like cardiovascular disease as well [2]. While there are numerous studies from western countries on physical activity levels in their respective populations, few studies from India have looked at this important risk factor. Moreover most of the available data have been derived from small studies conducted in discrete regions of the country, which have used varying methodology and have been conducted over different time periods [3-5]. Many of these studies also suffer from the problem of insufficient sample size and lack of proper representation from both urban and rural areas. The need for a representative nationwide survey on physical activity becomes all the more obvious when one considers the rapid economic and demographic transition that India is currently undergoing on account of economic liberalization, globalization and urbanization.

This paper reports on the levels of physical activity (and inactivity) in India, based on the results of Phase 1 of the Indian Council of Medical Research- India Diabetes (ICMR- INDIAB) study, which has studied a representative sample of three states and one union territory of India covering a population of about 213 million, and, which, to our knowledge, is the largest study on this subject from India.

## Methods

The study subjects were recruited from the ICMR-INDIAB study, a large ongoing cross-sectional, community based survey involving adults of both sexes aged 20 years and above. The study, when completed, will have sampled from all the 28 states of India, the National Capital Territory of Delhi and 2 union territories namely Chandigarh and Puducherry. In view of the complexity of the study and the logistics involved, the study has been planned and undertaken in phases. Phase 1 included 3 states namely Tamilnadu (population 67.4 million), Maharashtra (112.7 million) and Jharkhand (31.5 million) and one Union Territory namely Chandigarh (1.4 million) located in the south, west, east and north of the country respectively. INDIAB North East comprises the 8 North-Eastern states namely Assam, Arunachal Pradesh, Manipur, Meghalaya, Tripura, Sikkim, Mizoram and Nagaland and INDIAB Phase 2 involves the rest of the country. This paper deals only with Phase 1 of the study as the other Phases are still ongoing.

The methodology of the ICMR-INDIAB Study [6] and data on prevalence of diabetes from Phase 1 of the study [7] have been published earlier. Briefly, the sample size calculation was done based on previous estimates of the urban and rural prevalence of diabetes. Using a precision of 20% and allowing for a non-response rate of 20%, the sample size was calculated to be 4,000 per region (2,800 rural and 1,200 urban) [6]. In Phase 1, as we studied 4 regions, the overall sample size was calculated to be 16,000. A stratified multistage sampling design was adopted. The primary sampling units (PSUs) were villages in rural areas and census enumeration blocks in urban areas. Three-level stratification was done based on geography, population size and socio-economic status. A total of 16,607 individuals (5,112 urban and 11,495 rural) were selected from 363 PSUs (188 urban and 175 rural) of whom 14,277 individuals responded (response rate, 86%). Approval was obtained [from the Madras Diabetes Research Foundation Ethics Committee] prior to study commencement for all the states/UT and written informed consent was obtained from all participants in the local language.

For all participants, a structured questionnaire was administered to obtain data on socio-demographic parameters and behavioural aspects including physical activity. Physical activity was assessed using the Global Physical Activity Questionnaire (GPAQ), which has been developed by the World Health Organization (WHO) [8]. This questionnaire has 16 questions arranged in 3 main domains – occupation, travel and leisure activities. The major advantage of this questionnaire is that it can assess physical activity in each domain separately in addition to the total physical activity. GPAQ has been previously validated in 9 populations including Asian Indians and found to be reproducible and reliable [9]. Of the 14,277 subjects recruited in Phase I of the ICMR-INDIAB study, physical activity details were available for 14,227 subjects [Overall response rate, 99.6%; urban: n = 4,173, response rate, 99.7%; rural = 10054, response rate, 99.6%] who were included in the analyses.

Anthropometric parameters including height, weight and waist measurements were recorded using standardized techniques according to the Anthropometric Standardization Reference Manual [10]. Blood pressure was recorded using an electronic instrument (Model: HEM- 7101, Omron Corporation, Tokyo, Japan) as the mean of two readings taken five minutes apart.

In every fifth subject, a fasting venous sample was collected for measurement of lipids [serum cholesterol-cholesterol esterase oxidase-peroxidase-amidopyrine method; serum triglycerides -glycerol phosphate oxidase-peroxidase-amidopyrine method and HDL cholesterol- direct method-polyethylene glycol-pretreated enzymes] using the Beckman Coulter AU 2700/480 Autoanalyser [Beckman AU (Olympus), Ireland]. The intra- and inter-assay coefficients of variation for the biochemical assays ranged between 3 and 5%.

### Definitions used

#### Body mass index (BMI)

BMI was calculated using the formula, weight (in kilograms)/height (meters squared).

#### Socioeconomic status (SES)

SES for urban areas was determined by using the 2011 revised Kuppuswamy’s scale [11] of socio-economic status classification based on occupation, education and family income per month (in Rupees) as parameters. Individuals were classified as belonging to upper SES if the total score was 26–29, middle SES (upper middle and lower middle) if the total score was 11–25 and lower SES (upper lower and lower) if the total score was <11.

SES for rural areas was determined using house type and the Standard of Living Index (SLI) as given by the National Family Health Survey-3 (NFHS-3) [12]. Houses were classified as kachha, semi-pucca or pucca. In the SLI scoring system, facilities in the house, and possessions of the household were given scores. These scores were then summed-up and the result measured against a static set of SLI cut-offs. Households with a score 0–14 were classified as having a Low SLI, a score of 15–24 as Medium SLI and scores 25 and above were considered as High SLI.

#### Dyslipidemia

National Cholesterol Education Programme (NCEP) guidelines were used for definitions of dyslipidemia [13].

a. **Hypercholesterolemia:** serum cholesterol levels ≥200 mg/dl (≥5.2 mmol/liter) or on drug treatment for hypercholesterolemia.

b. **Hypertriglyceridemia:** serum triglyceride levels ≥150 mg/dl (≥1.7 mmol/liter) or on drug treatment for hypertriglyceridemia.

c. **Low High Density Lipoprotein (HDL) Cholesterol:** high-density lipoprotein cholesterol levels <40 mg/dl (<1.04 mmol/liter) for men and <50 mg/dl (<1.3 mmol/liter) for women.

d. **High Low Density Lipoprotein (LDL) Cholesterol:** low-density lipoprotein cholesterol levels ≥130 mg/dl (3.35 mmol/L) when calculated using the Friedewald equation [14].

#### Metabolic equivalents (MET)

MET is the ratio of a person’s working metabolic rate relative to the resting metabolic rate. One MET was defined as the energy cost of sitting quietly and was equivalent to a caloric consumption of 1 kcal/kg/hour [15].

#### Physical activity

To assess physical activity, MET scores were calculated separately for individual domains and sub domains, adopting existing guidelines [8].

When calculating a person's overall energy expenditure using GPAQ data, 4 METs were assigned to the time spent in moderate activities, and 8 METs to the time spent in vigorous activities. For the calculation of a categorical indicator, the total time spent on physical activity during a typical week, the number of days as well as the intensity of physical activity were taken into account. As per the guidelines for interpreting GPAQ Version 2.0, individuals were classified as active if, throughout a week (including activity for work, during transport and leisure time), they were involved in at least 150 minutes of moderate-intensity physical activity OR 75 minutes of vigorous-intensity physical activity OR an equivalent combination of moderate- and vigorous-intensity physical activity achieving at least 600 MET-minutes [8]. In addition, physical acitivity was further classified based on MET-minutes into three groups as: Inactive/low (<600 met-minutes), active (600–1200 met-minutes) and highly active (>1200 met-minutes).

### Statistical analysis

Statistical analyses were perfomed using a SAS (Statistical Analysis System) statistical package (version 9.0; SAS Institute, Inc., Cary, NC). For all stratification, the 2001 Census of India was used. Estimates were expressed as mean ± standard deviation or proportions. To compare continuous variable estimates between rural and urban areas,‘t’ tests were used, while chi square tests were used to test differences in rural–urban proportions within defined categorical variable groupings, respectively. One-way ANOVA (with post hoc Tukey -HSD procedure) was used to compare means of continuous variables between the three groups (inactive, active and highly active). For state projections, Government of India population projections for 2011 based on 2001 Census of India were used [16]. For national estimates, the data from the three states was used (The union territory was excluded as it may inflate projections). A p-value *<*0*.*05 was considered statistically significant.

## Results

Of the 14227 individuals studied, 54.4% (n = 7737) were inactive (male: 41.7%), while 31.9% (n = 4537) were active (male: 58.3%) and 13.7% (n = 1953) were highly active (male: 61.3%). The region-wise prevalence of physical inactivity was as follows: Chandigarh-66.8%, Tamilnadu-60.0%, Maharashtra- 55.2% and Jharkhand-34.9%. When extrapolated to the whole country, the estimated number of inactive individuals in India would be 392 million.

Table 1 shows the general characteristics of the study population stratified by physical activity levels. Inactive subjects were significantly older (p < 0.001), and had higher BMI (p < 0.001), waist circumference (p < 0.001), systolic (p < 0.001) and diastolic blood pressure (p = 0.003) and mean pulse rate (p < 0.001) compared to active and highly active subjects. They were also significantly less likely to smoke or consume alcohol than those in the other two groups. They were more likely to have an income above the median and belong to the upper socio-economic strata when compared to the other two groups. The inactive subjects also had significantly higher mean total cholesterol (p < 0.001) and triglyceride (p < 0.001) levels compared to the other two groups.

**Table 1:** General characteristics of the study population according to physical activity

**Variables**	**Inactive (n = 7737, 54.4%)**	**Active (n = 4537, 31.9%)**	**Highly active (n = 1953, 13.7%)**	**p value**
**Age (years)**	40.0 ± 15	40.0 ± 14	39 ± 13	<0.001
**Male n (%)**	3228 (41.7)	2645 (58.3)	1198 (61.3)	<0.001
**Body mass index (kg/m**^**2**^**)**	22.2 ± 4.5	21.2 ± 4.0	20.7 ± 3.8	<0.001
**Waist circumference (cms)**				
Male	81.6 ± 12.1	78.5 ± 11.6	77.5 ± 10.7	<0.001
Female	74.3 ± 12.4	72.6 ± 11.7	70.0 ± 10.7	<0.001
**Systolic BP (mm Hg)**	128.0 ± 19.0	127.0 ± 18.0	126 ± 17.0	<0.001
**Diastolic BP (mm Hg)**	78.0 ± 11.0	77.0 ± 11.0	77.0 ± 11.0	0.003
**Pulse rate**	79.0 ± 11.0	77.0 ± 11.0	76.0 ± 11.0	<0.001
**Alcohol n (%)**	1144 (14.8)	1078 (23.8)	535 (27.4)	<0.001
**Smoking n (%)**	999 (12.9 )	739 (16.3)	386 (19.8)	<0.001
**Income n (%)**				
Below median	2977 (42.0)	2770 (55.6)	1143 (62.7)	<0.001
Above median	4118 (58.0)	1813 (44.4)	679 (37.3)	
**Education n (%)**				
Illiterate	2213 (28.6)	1546 (34.1)	763 (39.1)	<0.001
Schooling	4736 (61.2)	2699 (59.5)	1116 (57.1)	
Under Graduates	683 (8.8)	251 (5.5)	68 (3.5)	
Post Graduates	99 (1.3)	37 (0.8)	5 (0.3)	
**Socio Economic status n (%)**				
Lower	859 (11.2)	754 (16.8)	422 (21.9)	<0.001
Middle	2035 (26.5)	1570 (35.0)	748 (38.8)	
Upper	4778 (62.3)	2166 (48.2)	758 (39.3)	
**Lipid profile (n = 2581)**	**(n = 1472)**	**(n = 784)**	**(n = 325)**	
Total cholesterol (mg/dl)	167.0 ± 39.0	154.0 ± 42.0	151 ± 36.0	<0.001
Triglycerides (mg/dl)^#^	152.0 ± 2.9	140.0 ± 4.0	135.0 ± 4.7	<0.001
HDL-cholesterol (mg/dl)				
Male (n = 1374)	37.0 ± 11.0	36.0 ± 11.0	37.0 ± 13.0	0.571
Female (n = 1207)	42.0 ± 12.0	39.0 ± 10.0	41.0 ± 13.0	0.179
Cholesterol/HDL ratio	4.6 ± 2.5	4.4 ± 2.4	4.2 ± 1.5	<0.001

^#^Geometric mean ± SE; Median income = Rs.3000/month.

Table 2 shows the physical activity levels in the four regions studied. Overall, in all the four regions studied, the prevalence of physical inactivity was significantly greater in urban areas compared to rural areas (65.0% vs. 50.0%; p < 0.001) and among females compared to males (63.0% [4509/7156] vs. 45.7% [3228/7071]; p < 0.001).

**Table 2:** Physical activity levels in the study population

**Physical activity**	**Rural**			**Urban**		
**Overall**						
	**Male (n = 5002)**	**Female (n = 5052)**	**Total (n = 10054)**	**Male (n = 2069)**	**Female (n = 2104)**	**Total (n = 4173)**
**Inactive n (%)**	2014 (40.3)	3011 (59.6)*	5025 (50.0)	1214 (58.7)^#^	1498 (71.2)*^@^	2712 (65.0)^$^
**Active n (%)**	2040 (40.8)	1407 (27.8)*	3447 (34.3)	605 (29.2)^#^	485 (23.0)*^@^	1090 (26.1)^$^
**Highly active n (%)**	948 (18.9)	634 (12.6)*	1582 (15.7)	250 (12.1)^#^	121 (5.8)*^@^	371 (8.9)^$^
**Chandigarh**						
	**Male (n = 1240)**	**Female (n = 1192)**	**Total (n = 2432)**	**Male (n = 444)**	**Female (n = 464)**	**Total (n = 908)**
**Inactive n (%)**	670 (54.0)	895 (75.1)*	1565 (64.4)	279 (62.8)^#^	386 (83.2)*^@^	665 (73.2)^$^
**Active n (%)**	379 (30.6)	224 (18.8)*	603 (24.8)	122 (27.5)^#^	62 (13.4)*^@^	184 (20.3)^$^
**Highly active n (%)**	191(15.4)	73 (6.1)*	264 (10.8)	43 (9.7)^#^	16 (3.5)*^@^	59 (6.5)^$^
**Jharkhand**						
	**Male (n = 1197**	**Female(n = 1187)**	**Total(n = 2384)**	**Male(n = 482)**	**Female(n = 463)**	**Total(n = 945)**
**Inactive n (%)**	165 (13.8)	525 (44.2)*	690 (28.9)	214 (44.4)^##^	256 (55.3)**^@^	470 (47.8)^$^
**Active n (%)**	732 (61.2)	460 (38.8)*	1192 (50.0)	205 (42.5)^#^	176 (38.01)^@^	381 (42.2)^$^
**Highly active n (%)**	300 (25.1)	202 (17.0)*	502 (21.1)	63 (13.1)^#^	31 (6.7)*^@^	94 (10.0)^$^
**Maharashtra**						
	**Male(n = 1313)**	**Female(n = 1343)**	**Total(n = 2656)**	**Male(n = 625)**	**Female(n = 623)**	**Total(n = 1248)**
**Inactive n (%)**	576 (43.9)	763 (56.8)*	1339 (50.4)	389 (62.2)^#^	428 (68.5)^@^	816 (65.4)^$^
**Active n (%)**	474 (36.1)	370 (27.6)*	844 (31.8)	159 (25.5)^#^	144 (23.1)^@^	303 (24.3)^$^
**Highly active n (%)**	263 (20.0)	210 (15.6)**	473 (17.8)	77 (12.3)^#^	52 (8.4)**^@^	129 (10.3)^$^
**Tamilnadu**						
	**Male(n = 1252)**	**Female(n = 1330)**	**Total(n = 2582)**	**Male(n = 518 )**	**Female(n = 554)**	**Total(n = 1072)**
**Inactive n (%)**	603 (48.2)	828 (62.3)*	1431 (55.4)	332 (64.1)^#^	429 (77.4)*^@^	761 (71.0)^$^
**Active n (%)**	455 (36.3)	353 (26.5)*	808 (31.3)	119 (23.0)^#^	103 (18.6)^@^	222 (20.7)^$^
**Highly active n (%)**	194 (15.5)	149 (11.2)**	343 (13.3)	67 (12.9)^#^	22 (4.0)*^@^	89 (8.3)^$^

*p <0.001 compared to male subjects; **p <0.05 compared to male subjects.

^$^p < 0.001 compared to rural residents; ^#^p <0.001 compared to rural male subjects; ^##^p <0.05 compared to rural male subjects; ^@^p <0.001compared to rural female subjects.

In Chandigarh, the prevalence of physical inactivity was significantly higher in urban compared to rural residents (73.2% vs. 64.4%; p < 0.001) and among females compared to males. There were more highly active subjects in the rural areas compared to the urban areas (10.8% vs. 6.5%; p < 0.001).

In Jharkhand, physical inactivity in urban areas was almost double of that seen in rural areas (47.8% vs. 28.9%; p < 0.001). In rural areas, significantly more males were classified as active compared to females (61.2% vs 38.8%; p < 0.001). However in urban areas these differences, although present, did not reach statistical significance. In both urban as well as rural areas, a significantly higher percentage of males were classified as highly active when compared to females (urban: male: 13.1% vs. female: 6.7%; p < 0.001; rural 25.1% vs. 17.0%; p < 0.001).

In Maharashtra also, the prevalence of physical inactivity was significantly higher in urban compared to rural areas (65.4% vs. 50.4%; p < 0.001). Interestingly, there was no significant difference in the prevalence of physical inactivity between males and females in urban areas. More rural males were classified as active compared to their female counterparts (male 36.1% vs female 27.6%; p <0.001). However, in urban areas, this difference did not reach statisitical significance. The percentage of those who were classified as highly active was significantly higher in rural compared to urban areas (17.8% vs. 10.3%; p < 0.001) and was significantly higher in males compared to females.

In Tamilnadu, a significantly greater proportion of urban residents were inactive compared to rural residents (71.0% vs. 55.4%; p < 0.001), while a significantly higher proportion of rural subjects were classified as highly active compared to their urban counterparts (13.3% vs. 8.3%; p < 0.001). Compared to males, a higher proportion of female subjects were physically inactive in both the urban as well as rural areas. Conversely, a higher proportion of males were found to be highly active as compared to females, in both the urban as well as rural areas.

Table 3 shows the average minutes spent in moderate to vigorous intensity physical activity per day in the various activity domains namely work, transport and recreation in all the regions studied.. Overall, it was found that most of the time spent in moderate to vigorous intensity activity was at the workplace, with individuals reporting an average of 46 minutes of moderate to vigorous intensity activity per day at work. This was highest in Jharkhand followed by Maharashtra, Chandigarh and Tamilnadu in that order. Males spent more time in moderate to vigorous activity at the workplace compared to females across all the regions studied.

**Table 3:** Mean minutes of moderate to vigorous intensity activity per day in the various domains in all the regions studied

**State/UT**		**Chandigarh**	**Jharkhand**	**Maharashtra**	**Tamilnadu**	**Overall**
**Overall**						
**Work**	**n**	**683**	**1175**	**1238**	**1331**	**4427**
(min/day)	**Mean**	44.3	51.3	50.8	37.1	45.8
	**SE**	1.04	1.71	0.70	0.75	0.57
**Transport**	**n**	**2808**	**3025**	**3451**	**3160**	**12444**
(min/day)	**Mean**	12.8	20.4	12.4	12.2	14.3
	**SE**	0.14	0.22	0.12	0.13	0.08
**Recreation**	**n**	**294**	**120**	**270**	**192**	**876**
(min/day)	**Mean**	20.5	28.9	16.21	15.2	19.2
	**SE**	0.81	2.23	0.59	1.16	0.53
**Male**						
**Work**	**n**	**586**	**500**	**667**	**593**	**2346**
(min/day)	**Mean**	45.1	72.0	57.0	51.2	55.8
	**SE**	1.14	3.5	1.04	1.12	0.92
**Transport**	**n**	**1487**	**1573**	**1747**	**1607**	**6414**
(min/day)	**Mean**	11.7	25.7	12.9	12.7	15.7
	**SE**	0.18	0.3	0.17	0.19	0.12
**Recreation**	**n**	**194**	**112**	**200**	**147**	**653**
	**Mean**	19.6	29.7	16.5	15.9	19.6
	**SE**	1.03	2.4	0.73	1.46	0.67
**Female**						
**Work**	**n**	**97**	**675**	**571**	**738**	**2081**
(min/day)	**Mean**	39.8	36.1*	43.5*	25.7*	34.6*
	**SE**	2.43	1.08	0.80	0.78	0.53
**Transport**	**n**	**1321**	**1452**	**1704**	**1553**	**6030**
(min/day)	**Mean**	14.0*	14.2*	11.9*	11.6*	12.8*
	**SE**	0.20	0.29	0.17	0.18	0.11
**Recreation**	**n**	**100**	**8**	**70**	**45**	**223**
(min/day)	**Mean**	22.2	17.1	15.3	12.7	18.0
	**SE**	1.26	3.06	0.96	1.3	0.75

*p <0.001 compared to male subjects.

Overall, subjects reported spending a mean of only 14 minutes per day on moderate to vigorous intensity activity in the transport domain. This, again was highest in Jharkhand with the other three regions reporting lower but similar levels. Except in Chandigarh, males spent significantly more time doing moderate to vigorous intensity activity in this domain compared to females.

**Figure 1:**
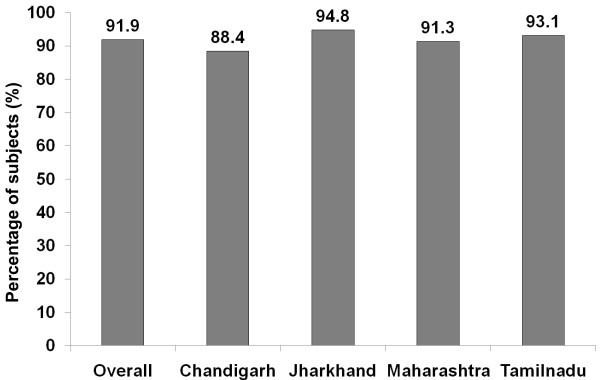
State wise distribution of subjects with no recreational activity.

**Figure 2:**
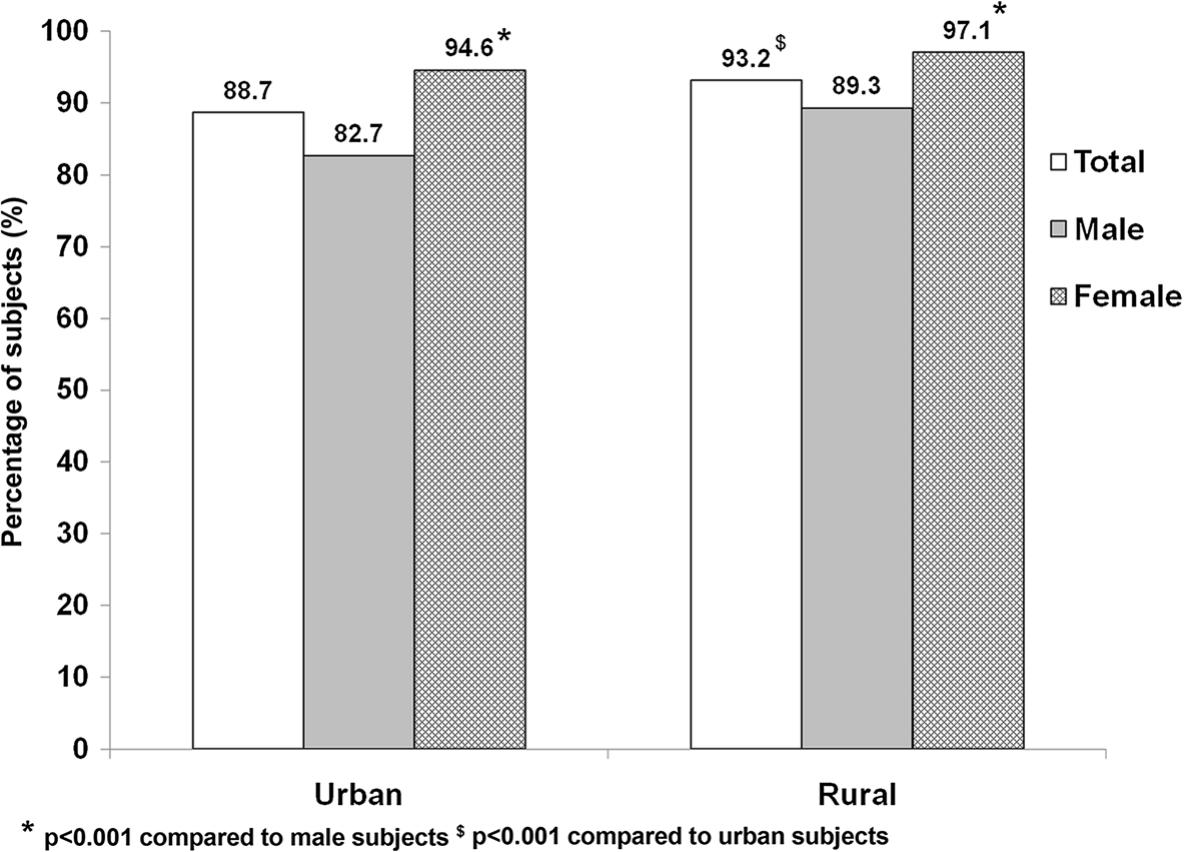
Gender and area wise distribution of subjects with no recreational activity.

In the recreational domain, it was found that subjects spent less than 20 minutes per day in moderate to vigorous intensity activity. This was again highest in Jharkhand, followed by Chandigarh, Maharashtra and Tamilnadu. Except in Chandigarh, males spent more time doing moderate to vigorous intensity activity in this domain compared to females. However none of these differences reached statistical significance.Figure 1 illustrates the percentage of subjects reporting no recreational activity in the four regions studied. Overall, 91.9% of the subjects in the four regions studied did not do any recreational activity [88.4% in Chandigarh, 94.8% in Jharkhand, 91.3% in Maharashtra and 93.1% in Tamil Nadu]. Figure 2 illustrates the gender and area wise distribution of subjects who reported no recreational activity. A significantly higher proportion of rural subjects performed no recreational activity as compared to urban subjects (93.2% vs. 88.7%, p < 0.001). In both urban and rural areas, a significantly higher proportion of females reported no recreational activity compared to males (Urban: 94.6% vs. 82.7%, p < 0.001; Rural: 97.1% vs. 89.3%, p < 0.001).

**Figure 3:**
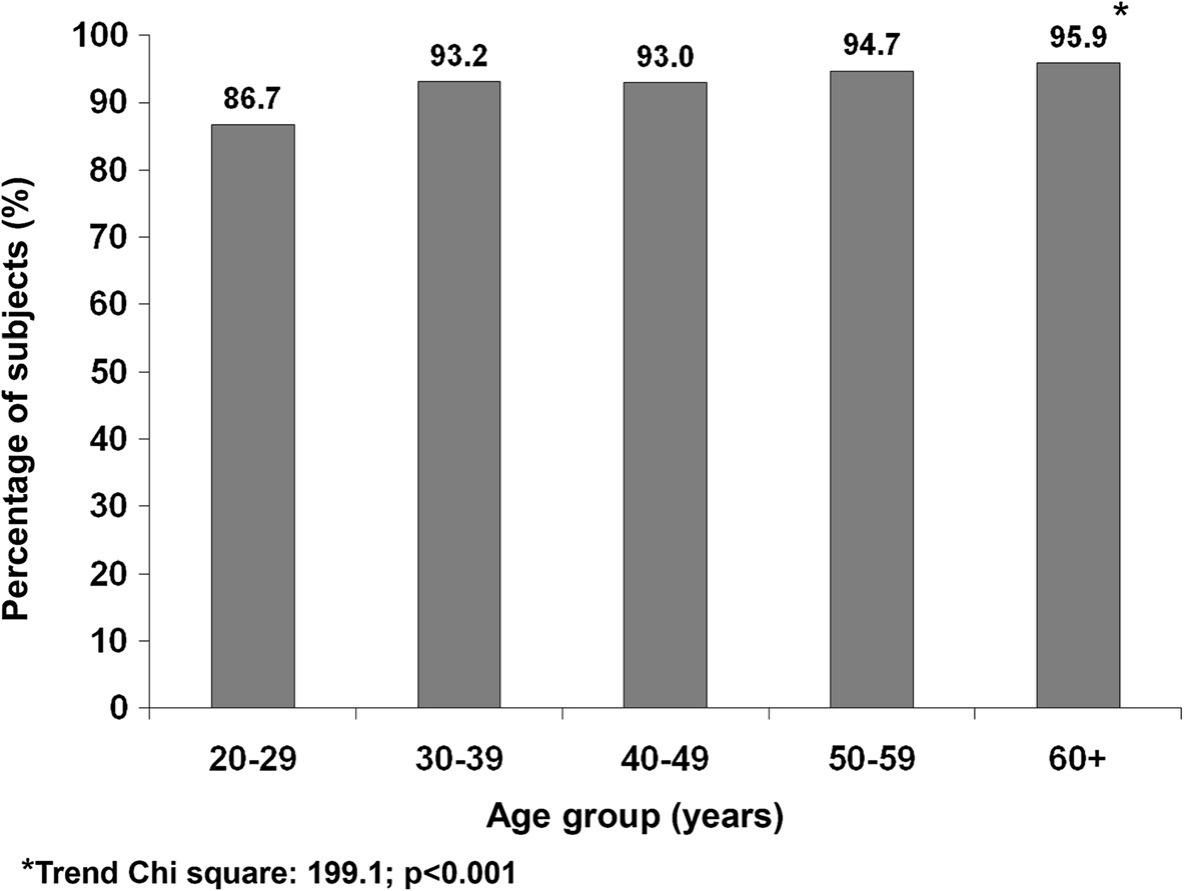
Age wise distribution of subjects with no recreational activity in the population.

Figure 3 shows the percentage of subjects with no recreational activity in different age groups. The proportion of subjects reporting no recreational activity steadily increased with increasing age from 86.7% to 95.9%. (Trend *χ*^2^: 199.1, p < 0.001).

## Discussion

This is the largest study till date to report on physical activity patterns in India. Our results show the following:

•Nearly half of the population in the four regions studied were inactive. This translates to 392 million inactive individuals in India.

•Physical inactivity was significantly more common in urban areas of the country compared to rural areas

•Males were significantly more active than females

•Most of the time spent in moderate to vigorous intensity activity was at the workplace.

•More than 90% of subjects in all the four regions studied did no recreational physical activity. This was significantly higher in rural areas and among females.

•Even among those who reported recreational physical activity, the time spent in moderate to vigorous intensity activity was overall less than 20 mins/day.

Over the past decade, a number of studies carried out in developed nations have shown high prevalence of physical inactivity. The Behavioral Risk Factor Surveillance System [17] published in 2003 showed that 52.8% of U.S. citizens were inactive (50.2% men and 55.4% women). In a study done using the International Physical Activity Questionnaire (IPAQ) in Sweden in 2002–03 on 1470 adults aged 18 to 74 years, 31% of the population was found to be inactive [18] while the Health Survey for England [19] reported a 63% prevalence of inactivity for men and 76% prevalence for women. The Eurobarometer wave 58.2 [20] showed a combined prevalence of inactivity of 31%. However, the 51 country study of worldwide variability in physical inactivity [World Health Survey (WHS)] [21], showed overall physical inactivity to be much lower (17.7%; 19.8% women and 15.2% men). In the WHS, the prevalence of physical inactivity in India was 9.3% in men and 15.2% in women. This figure is much lower than those seen in the present study (54.4%; 41.7% men). However, the WHS was performed more than a decade ago, and India has gone through far reaching demographic and socioeconomic changes in the interim, making comparisons of that survey with the present study difficult. Also, the WHS used the IPAQ, whereas in the present study we have used the GPAQ, which could account for some of the differences seen [22].

A 20 country study [23] conducted between 2002 to 2004 showed that the prevalence of "low physical activity" varied from 9% to 43%. A review of 55 population based sureys of physical activity from 29 Asia-Pacific countries was published recently [24]. Varying methodologies were used in the different studies – 19 surveys used the IPAQ, 18 surveys used the GPAQ and 18, other instruments. The review demonstrated that physical activity estimates vary widely even within a single country using different surveys in similar time periods. Three surveys from India were included in the above review- the WHO Modified STEPS Survey (GPAQ) in 2003–2005, the World Health Survey (IPAQ) in 2003 and the IPAQ Short Form (2003). The prevalence rates of “sufficiently active” were 84, 88 and 77% respectively. These figures are much higher than that shown in this study, which could be accounted for by the different methodology and sample selection criteria adopted by these studies. It is also possible that over the last decade, physical activity levels may have considerably declined, although such wide differences probably reflect methodological variations.

Indeed, a more recent multisite cross sectional study done in 9 rural areas in five Asian countries utilizing the GPAQ showed that levels of physical inactivity varied from 13% to 58%. In the study site in India (Vadu, Maharashtra), the level of physical inactivity was 53% [25], which is similar to that in our study (50.0%).

A study done by Shah et al., [26] in 2005 in six regions of India (Delhi and Ballabgarh in the north, Chennai and Trivandrum in the south, Nagpur in the west and Dibrugarh in the east) showed that overall inactivity levels were 12.6% in males and 18.9% in females. Moreover, this study also showed marked variability in physical activity in different regions of the country. In the current study also, there was variability in physical activity across different regions of the country; however, there seems to be a marked increase in the proportion of individuals reporting inactivity compared to the study conducted eight years ago. This could again point to declining physical activity levels in recent times.

A more recent study done using cluster sampling in 6198 subjects (3426 men and 2772 women) from eleven cities across India showed that 38.8% of men and 46.1% of women were physically inactive [27], and these figures are similar to those reported in the present study. Similarly, another recent study from Jaipur looked at the prevalence of cardiovascular risk factors in 739 subjects (451 men, 288 women). It was found that 69.6% of men and 52.4% of women were physically inactive [28].

Hallal et al. [29] in a recent review showed that the prevalence of physical inactivity varied widely between regions of the world: 27.5% in Africa, 43.3% in the Americas, 43.2% in the Eastern Mediterranean, 34.8% in Europe, 17% in South East Asia and 33.7% in the Western Pacific.

We report that the highest prevalence of physical inactivity was found in Chandigarh (66.8%). This is not surprising since Chandigarh is a highly urbanized territory and is located adjacent to two of India’s prosperous states, Punjab and Haryana, of which it serves as the joint capital. This can also explain why the rural urban disparity in inactivity is the least marked in Chandigarh. In a study conducted in 2010 on 2227 subjects aged 20 years and above in a representative sample of the urban Chandigarh population, Ravikiran et al. [30] found that 61.3% of the study subjects were inactive, which is similar to our results.

We found that there was a significant difference among males and females with respect to physical activity, with males being more active. This is in agreement with earlier studies, most of which have reported higher levels of activity in males compared to females [18,20,26,29,31].

The prevalence of inactivity was higher in urban areas compared to rural areas. Factors like higher levels of income, less physically demanding occupations and increased availability of mechanized transport and household appliances among urban dwellers could explain this disparity. A similar finding was noted in a study from Tamilnadu [32], which found that levels of sedentary behaviour were highest in the cities, followed by the smaller towns and the periurban villages. This also agrees with the findings of the 51 Country Study quoted above [21]. A similar finding was also noted in China, where the percentage of active individuals was found to be much higher in the rural areas compared to the urban areas (78.1% vs. 21.8%) [33].

Our study underscores the fact that leisure-time or recreational physical activity levels are extremely low in India. More than 90% of individuals in both urban and rural areas reported doing no recreational physical activity. This is similar to the situation in China [33], where only 28.9% of rural residents and 7.9% of urban residents reported leisure time physical activity. In Brazil also, the prevalence of physical inactivity in the recreational domain was found to be 80.7% [34]. Another study from Vitenam also reported inactivity in the recreational domain to be as high as 90.6%, which is similar to our findings [35]. The high prevalence of insufficient recreational activity observed across all age groups and both genders could reflect limited access to and availability of facilities for recreational physical activity.

The WHO recommends that individuals perform at least 150 minutes of moderate to vigorous physical activity per week for the maintenance of health. In India at the present time, more than half of the population do not meet these recommendations. Moreover, individuals appear to derive most of their physical activity from the occupational domain. This is similar to the situation in China and Vietnam, where most of time spent in physical activity is in the work domain [35,36]. As physical activity levels in the occupational domain decline, individuals will have to obtain much of their physical activity requirements through their leisure time pursuits. This assumes significance in view of our findings that over 90% of the population do no recreational physical activity at all. This points to the need for increasing awareness regarding physical activity in India, and provision of facilities for individuals in both urban and rural areas to engage in recreational physical activity.

There are some limitations to this study. For assessment of physical activity, GPAQ has been used, which has been designed chiefly for surveillance purposes in developing countries. As for any self-reporting measure, recall bias leading to over- or under-reporting of physical activity cannot be ruled out in the study. Moreoever, the GPAQ may not be culturally specific. In the present study, data from three states of India has been used to calculate the numbers of physically active persons in the country. It is possible that these numbers might change once the entire study, comprising all the states of the country, is completed. However, since the three states have been selected to represent distinct regions of the country and within each region, a representative population has been sampled in both the urban and rural areas major changes in the numbers are unlikely.

## Conclusions

The results show that overall 392 million individuals are inactive in India. This is a staggering figure and implies a huge population at risk for developing diabetes and other non communicable diseases. This underscores the urgent need to improve overall physical activity levels with specific reference to recreational physical activity. This could go a long way in curtailing the twin epidemics of diabetes and obesity in India.

## Abbreviations

ICMR-INDIAB study: Indian Council of Medical Research- India Diabetes Study; WHO: World Health Organization; GPAQ: Global physical activity questionnaire; PSU: Primary sampling unit; BMI: Body mass index; SES: Socioeconomic status; NFHS-3: National Family Health Survey; HDL: High density lipoprotein; LDL: Low density lipoprotein; MET: Metabolic equivalents; IPAQ: International Physical Activity Questionnaire; WHS: World Health Survey.

## Competing interests

The authors declare that they have no competing interests.

## Authors’ contribution

RMA and VM conceived the study, its design, and were involved in implementation and execution of the study, interpretation of the data and drafted and revised the manuscript. RP, MD, SV and RU were involved in the design and coordination of the study, helped in the execution of the study, were responsible for maintaining quality in the study, interpretation of the data and helped in drafting the manuscript. AB, SRJ, PPJ, and VKD were responsible for supervision of the study in their respective states. RP, SS and RS were responsible for data management and statistical analysis. AKD, PVR, SVM, DKS and TK were part of the study Expert Committee and helped with the conception and design of the study and revising the manuscript critically for important intellectual content. All authors read and approved the final manuscript.
